# Developmental validation of the AGCU YNFS Y Kit: A new 6-dye multiplex system with 44 Y-STRs and 5 Y-InDels for forensic application

**DOI:** 10.1371/journal.pone.0308535

**Published:** 2024-08-09

**Authors:** Chaoran Sun, Xindi Wang, Shuangshuang Wang, Yuxiang Zhou, Lanrui Jiang, Zefei Wang, Hewen Yao, Zhirui Zhang, Lagabaiyila Zha, Haibo Luo, Feng Song

**Affiliations:** 1 Department of Forensic Genetics, West China School of Basic Medical Sciences & Forensic Medicine, Sichuan University, Chengdu, Sichuan Province, China; 2 Department of Forensic Science, School of Basic Medical Sciences, Central South University, Changsha, Hunan Province, China; Xiamen University, CHINA

## Abstract

With the widespread use of the Y chromosome in genetics, a lot of commercially available Y chromosome kits were developed, validated, and applied to forensic science practice. The AGCU YNFS Y Kit is a new Y chromosome system containing forty-four preferred Y short tandem repeats (Y-STRs) and five common Y-InDels. In this study, the AGCU YNFS Y system was validated to verify its performance by following the guidelines of the Scientific Working Group on DNA Analysis Methods (SWGDAM). A series of validation experiments included the following parameters: PCR-based studies, sensitivity studies, species specificity studies, stability studies, mixture studies, precision studies, stutter calculation, mutation and statistical analysis, population study, and case samples and degradation studies. The results suggested that appropriately changing PCR amplification conditions did not affect genotyping; the kit had good sensitivity for trace amounts of DNA (0.0625 ng), mixtures of multiple male individuals (minor: major = 1: 9), and three PCR inhibitors (more than 250 μM hematin, 250 ng/μL humic acid and 50 ng/μL tannic acid). The maximum standard deviation of allele size did not exceed 0.1552 reflecting the high accuracy of the system. By this, 87 DNA-confirmed pairs of father-son pairs were also analyzed for mutations. A total of 18 loci were mutated, with mutation rates ranging from 11.5×10^−3^ to 34.5×10^−3^ (95% CI 7.2×10−3–97.5×10^−3^, DYS627 and DYF404S1). In the population study, the haplotype diversity of 87 unrelated individuals was 0.9997, and discrimination capacity was 0.9885. Degradation studies have demonstrated that UV-C light exposure for up to 120 hours has no effect on male blood and semen-vaginal secretion mixtures. However, complete typing could no longer be obtained after 48 hours of UV exposure in single male saliva and in male saliva and female blood mixed samples. Collectively, the AGCU YNFS Y Kit is sensitive and accurate and can play its application value in forensic science practice.

## 1. Introduction

The Y chromosome is a male-specific sex chromosome and can be divided into two regions according to different modes of inheritance. One is the pseudoautosomal region (PAR) located at both ends of the Y chromosome, which can recombine with the corresponding region of the X chromosome, and the other is the non-recombining Y (NRY), which is the male-specific region (MSY) [1, 2]. Short tandem repeats (STRs) are still the gold standard for individual identification and kinship analysis in forensic medicine due to their high polymorphism, heterozygosity, and relative stability [3]. Y-chromosomal short tandem repeats (Y-STRs) are characterized by paternal and haplotype inheritance, and the haplotypes of male individuals in the same paternal line are identical in the absence of mutations. The Y-STR test has important application value for paternity identification, family investigation, male component detection of mixed spots, and ethnic origin inference [4].

Currently, the application of the Y-STR construction database to conduct family line searches and trace suspicious family lines helps to indicate the direction of the investigation and narrow down the range of suspects. However, distinguishing between different male individuals within the same paternal line remains one of the goals pursued in the field of forensic genetics. Adding more types and numbers of Y-chromosome genetic markers is expected to solve the above problem. It has been shown that Y-STRs with high mutation rates have the potential to differentiate male relatives of the same paternal line [5]. In a 2010 study analyzing mutation rates of Y-STRs, Ballantyne et al. identified 13 rapidly mutating Y-STRs (RM Y-STRs) with mutation rates of up to 10^−2^/generation, which provided a powerful tool for distinguishing paternal male relatives [6]. Ralf et al. discovered 12 new RM Y-STRs in their 2020 study [7]. Thereafter, RMplex, a system that integrates 26 RM Y-STRs and 4 fasting mutating Y-STRs (FM Y-STRs), was invented and introduced into forensic genetics [8]. By combining a lot of Y-STRs with high mutation rates, it is expected that the discrimination of male relatives from the same paternal line will be realized, allowing the identification of criminal suspects within the family line. Moreover, in recent years, insertion-deletion (InDel) markers have gradually gained attention due to their advantages such as low mutation rate and small amplified fragments [9]. InDel is widely distributed in the human genome, with one InDel in about 7.2 kb [10]. Based on these, InDel has a better ability to analyze highly degraded DNA samples and events in which STR is mutated in paternity testing [11, 12]. Y-InDel can be mainly used as an auxiliary marker for sex determination and in combination with Y-STR as a supplement.

**Fig 1:**
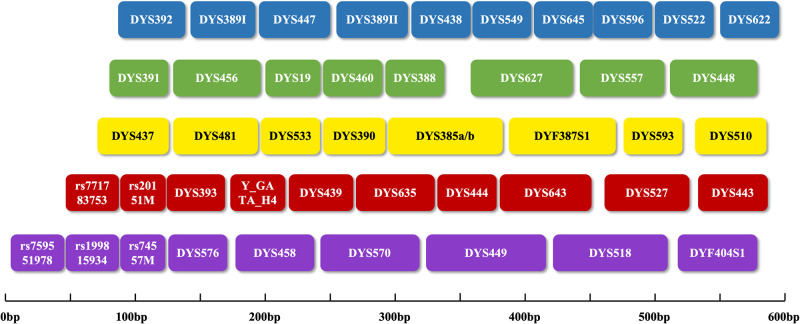
Size and range of loci in the AGCU YNFS Y Kit.

Faced with the need to solve the problem and combine the advantages of Y-STR and Y-InDel, currently, a new amplification system called the AGCU YNFS Y Kit (AGCU ScienTech Incorporation, Wuxi, Jiangsu, China), employs a six-dye fluorescent label with a total of 44 Y-STRs and 5 common Y-InDels, which contains most of the Y-STRs appearing in the kits that have been applied to in practical cases. Among these loci, in addition to the size standard fluorescence, the blue channel includes DYS392, DYS389I, DYS447, DYS389II, DYS438, DYS549, DYS645, DYS596, DYS522 and DYS622. The green channel contains DYS391, DYS456, DYS19, DYS460, DYS388, DYS627, DYS557, and DYS448. The yellow channel comprises DYS437, DYS481, DYS533, DYS390, DYS385, DYF387S1, DYS593 and DYS510. The red channel incorporates rs771783753, rs20151M, DYS393, Y-GATA-H4, DYS439, DYS635, DYS444, DYS643, DYS527 and DYS443. The purple one encompasses rs759551978, rs199815934, rs74557M, DYS576, DYS458, DYS570, DYS449, DYS518 and DYF404S1. The size and range of each locus are shown in Fig 1.

A series of validation experiments were conducted to evaluate the efficiency of this new kit, containing PCR amplification conditions, sensitivity, stability, species specificity, precision and accuracy, mixture testing, mutation analysis, population study, and case samples and degradation studies. The developmental validation of the kit followed the guidelines of the Scientific Working Group on DNA Analysis Methods (SWGDAM) [13].

## 2. Materials and methods

### 2.1. Sample preparation

A total of four standard control DNA samples, which encompass 9948 (Thermo Fisher Scientific, Waltham, MA, USA), 007 (Thermo Fisher Scientific, Waltham, MA, USA), M308 (Beijing Microreader Genetics, Beijing), and K562 (Promega, Madison, WI, USA), were used in this study. The first three were male samples, while K562 was a female sample. Nine animal species including cavies, goats, cats, dogs, chickens, ducks, rabbits, pigs, and cattle were selected to perform species studies (Zyagen, CA, USA) [14]. Blood samples from 87 DNA-confirmed father-son pairs were collected from the Han Chinese population in Sichuan Province, China and the Chelex 100 method was used to extract the DNA from father-son pairs [15]. Additionally, saliva and blood samples from three males, semen samples from two males, and blood and vaginal secretion samples from one female were collected for forensic scene simulation and degradation studies. DNA was obtained from these samples using the QIAamp^®^ DNA Mini Kit (QIAGEN, Germany), as supplied by the manufacturer’s protocol. All participants provided their written informed consent and sample collection was approved by the Medical Ethics Committee of Sichuan University (K2019018). The recruitment period for our study started on 1 January 2021 and ended on 31 June 2024.

### 2.2. DNA amplification

If not specifically declared, PCR amplification was strictly followed according to the instructions of the AGCU YNFS Y Kit. The total reaction was carried out in a 0.2 ml tube with a total volume of 25 μL, which consisted of 10 μL of YNFS Y Mix Pro, 5 μL YNFS Y Primers Pro, and 0.5 ng of template DNA, and then nuclease-free water was added to 25 μL. The specific steps for standard thermal cycling conditions are as follows. The initial denaturation step was a preincubation at 95 °C for 2 min, subsequently followed by 28 cycles of denaturation at 94 °C for 30 s, annealing at 60 °C for 1 min, and extension at 66 °C for 1 min. Eventually, the final extension step was performed at 60 °C for 20 min and kept at 4 °C for further analysis.

### 2.3. Capillary electrophoresis and data analysis

The AGCU YNFS Y Kit uses the six-dye chemistry, which includes FAM, HEX, SUM, LYN, PUR, and SIZ, providing YNFS Y Allelic Ladder and AGCU Marker SIZ-600. It is necessary to establish the spectral resolution using the A6Dye Matrix for correcting each fluorescent dye in this kit. Additionally, all PCR products were detected by the Applied Biosystems 3500 Genetic Analyzer (Thermo Fisher Scientific, Waltham, MA, USA) with a 36 cm capillary array and POP-4 polymer. The specific reaction conditions for 3500 are as follows. Samples were injected at 1.2 kV for 10 s and electrophoresed at 15 kV for 1310 s at a run temperature of 60 °C. 1 μL of PCR products or allelic ladder was added to a 9 μL mixture of Hi-Di formamide and AGCU Marker SIZ-600. GeneMapper ID-X1.5 Software (Applied Biosystems Foster City, CA) was utilized to analyze the raw data with 50 relative fluorescence units (RFU) as the analytical threshold for peak height.

### 2.4. PCR-based studies

There are some key parameters of PCR amplification conditions. It is significant to evaluate the performance of key PCR parameters and determine the parameter ranges of PCR amplification conditions for obtaining accurate and stable STR genotyping results. This study tested the following five parameters containing PCR reaction volume (25 μL, 15μL, 10 μL, 5μL, 3.5μL), cycle numbers (22 cycles, 24 cycles, 26 cycles, 28 cycles, 30 cycles, 32 cycles, 34 cycles), annealing temperature (56 °C, 58 °C, 60 °C, 62 °C, 64 °C), the volume of YNFS Y Mix Pro (6 μL, 8 μL, 10 μL, 12 μL, 14 μL) and the volume of YNFS Y Primers Pro (1 μL, 3 μL, 5 μL, 7 μL, 9 μL). 9948 DNA was employed as template DNA. 0.5 ng DNA was used in a 25 μL PCR reaction volume, 0.3 ng DNA in a 15 μL volume, 0.2 ng DNA in a 10 μL volume, 0.1 ng DNA in a 5 μL volume, and 0.07 ng DNA in a 3.5 μL volume. While the other conditions were identical to the recommended conditions, the test condition was changed sequentially. A series of PCR-based studies were tested in triplicate.

### 2.5. Sensitivity studies

Sensitivity experiments were performed to assess the performance of the kit using 9948 DNA. The total DNA inputs tested, which ranged from 0.03125 ng to 2 ng, were 0.03125 ng, 0.0625 ng, 0.125 ng, 0.25 ng, 0.5 ng, 1 ng, and 2 ng in a volume of 25 μL. Male control 9948 DNA was diluted with nuclease-free water and amplified in triplicate.

### 2.6. Species specificity studies and stability studies

It is necessary to consider the possibility of other biological sources at the crime scene. DNA from nine common animals was used to evaluate the species specificity of the AGCU YNFS Y Kit, including dogs, cats, chickens, ducks, rabbits, pigs, cattle, goats, and cavies. All DNA samples were prepared at 2 ng for further amplification and analysis.

Since samples obtained in forensic practice usually contain some PCR inhibitors, we validated the anti-interference capability of the kit with three different PCR inhibitors. They were hematin at concentrations of 125 μM, 250 μM, 500 μM and 1000 μM; humic acid at concentrations of 250 ng/μL, 500 ng/μL, 750 ng/μL, 1000 ng/μL and 1500 ng/μL; and tannic acid at concentrations of 50 ng/μL, 75 ng/μL, 100 ng/μL and 125 ng/μL. 0.1 ng of the control 9948 DNA was held constant in a 5-μL volume and amplified with the PCR inhibitors under the protocol of the AGCU YNFS Y Kit.

### 2.7. Mixture studies

Mixture experiments will investigate the gender-specificity of the AGCU YNFS Y Kit in a context with female DNA. First of all, female DNA K562 remained unchanged at a concentration of 400 pg. Secondly, the study used three different male samples, and added two male and male DNA mixtures with a total input volume of 1 ng to the constant 400 pg female standard in proportion, with the ratios being 1: 1, 3: 1, 9: 1 and 19: 1. Male control 9948 DNA was mixed with 007 DNA and M308 DNA respectively.

### 2.8. Precision study and stutter calculation

A total of 45 allele ladders were used for precision analysis of this kit, which was performed 24 injections on an Applied Biosystems 3500 Genetic Analyzer. We calculated the average size and standard deviation (SD) of each allele to measure the precision [16]. The stutter ratio was gained by dividing the peak height of the stutter peak by the peak height of the major allele, with the analytical threshold of the minimum stutter peak height set at 20 RFU.

### 2.9. Mutation analysis and population study

Data from the 87 father-son pairs were analyzed for mutation analysis studies of the AGCU YNFS Y Kit. The data obtained were used to calculate the mutation rate per generation for each locus, which is the ratio between the number of mutation events at that locus and the total number of alleles transferred from father to son. The binomial 95% confidence interval (CI) for the mutation rate was evaluated on the website (https://statpages.info/confint.html).

The population study was used to demonstrate the forensic genetic character of the AGCU YNFS Y Kit, and 87 unrelated individuals were subject to population genetics studies. The following forensic parameters, including allele frequencies, haplotype frequencies, fraction of unique haplotype (FUH), haplotype diversity (HD), gene diversity (GD), and discrimination capacity (DC), were calculated using StatsY v1.0 [17].

### 2.10. Case samples and degradation studies

We collected saliva, blood, and semen samples from males and blood and vaginal secretions from one female, which are common in forensic cases, to prepare simulated samples. Firstly, single samples were prepared by taking 100 μL drops of saliva and blood from each of the three males onto a sterile cotton swab. Secondly, the saliva samples of the three males were mixed with the blood samples of the females, and the semen of the two males and vaginal secretions were mixed for the preparation of mixed male and female samples, respectively. Thus, a total of 3 sets of male blood samples, 3 sets of male saliva samples, 3 sets of male saliva and female blood mixtures, and 2 sets of male semen and female vaginal secretion mixtures were prepared. For subsequent experiments, the above samples were taken in triplicate. To investigate the stability of the AGCU YNFS Y Kit in degraded samples, we tested these samples kept in UV-C light exposure for 0 hours, 24 hours, 48 hours, 72 hours, 96 hours, and 120 hours. Thus, this part of the study had a total of 66 samples. UV-C light exposure was followed by DNA extraction, amplification in triplicate, and electrophoresis.

## 3. Results and discussion

### 3.1. PCR-based studies

**Fig 2:**
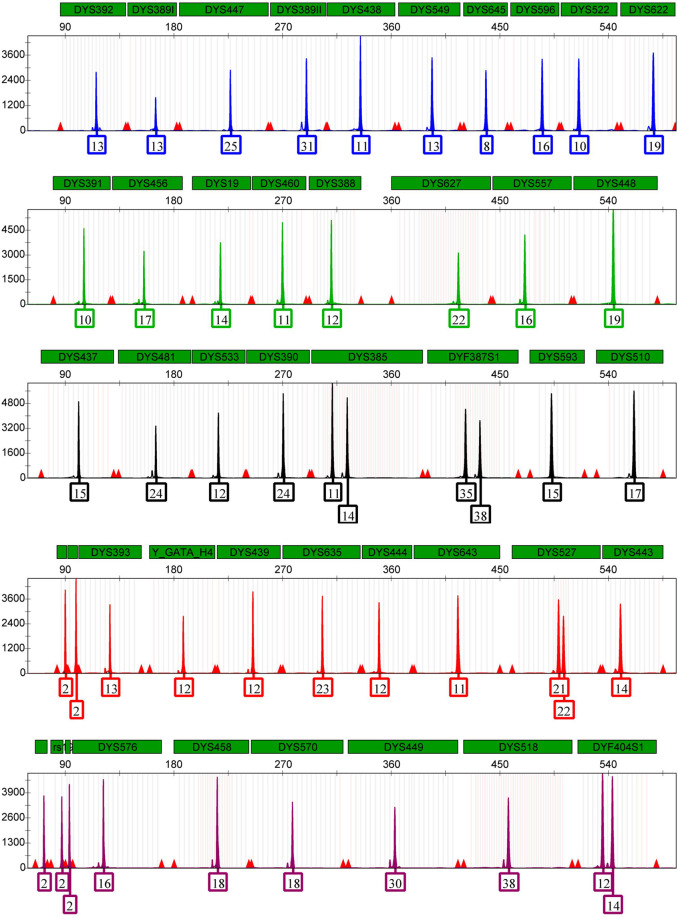
The complete typing profile of control 9948 DNA. The total reaction was carried out with a total volume of 25 μL, which consisted of 10 μL of YNFS Y Mix Pro, 5 μL YNFS Y Primers Pro, and 0.5 ng of template DNA, and then nuclease-free water was added to 25 μL.

The standard PCR reaction volume for this system was 25 μL and the complete typing profile of control 9948 DNA was shown in Fig 2. Considering the analysis of forensic trace material analysis and the smaller volume is more suitable for Low Copy Number DNA samples, it is significant to reduce the reaction volume by the same proportion to save reagents and reduce costs. When dealing with samples with low template DNA (LTDNA), the sensitivity and accuracy of STR analysis were often improved by reducing the reaction volume [18, 19]. Complete typing profiles without allelic deletions were detected in five different PCR reaction volumes ranging from 3.5 μL to 25 μL by using control 9948 DNA (S1 Fig). The results showed that accurate genotyping results could still be obtained with a reaction volume of only 3.5 μL, which demonstrated the excellent performance of the AGCU YNFS Y Kit in handling Low Copy Number DNA samples in a small volume.

Other four parameters of PCR amplification conditions also play an important role in PCR amplification [20]. In the experiment exploring the number of PCR cycle numbers, the results demonstrated that the more the number of PCR cycles, which ranged from 22 to 34 cycles, the higher the peak height (S2 Fig). Genotyping was complete when the number of cycles was 24, 26, 28, 30, 32. However, when the number of cycles was 22, too low, no correct peaks were detected for any locus. As for 34 cycles, lots of cleft peaks and spike peaks appeared on the motifs with smaller fragments, and the vast majority of motifs showed more stutter peaks and non-specific peaks, which did not allow for accurate genotyping. The cause of this phenomenon may be the high concentration of PCR products resulting in too many cycles. Given that the recommended number of PCR cycles of this system is 28, and that the genotyping results are complete at 24–32 cycles, the number of cycles can be reasonably adjusted according to the actual situation of samples and time in forensic practice. In the study on annealing temperature (S3 Fig), when the annealing temperature ranged from 56 to 62 °C, we could observe full profiles. While at 64 °C, loci including DYS447, DYS549, DYS19, DYS437, DYS385, rs759551978 dropped out. The results on annealing temperature suggested that the AGCU YNFS Y Kit responded well to small deviations in annealing temperature so that the probability of failure due to inaccurate temperature was reduced.

Changes in the concentration of PCR components can affect the efficiency of amplification and the appearance of non-specific products [21]. Experimental manipulation errors may result in the wrong concentration of PCR components being added. In this study, completely correct typing results were detected and no allele dropped out with the condition of 6–14 μL YNFS Y Mix Pro and 3–9 μL YNFS Y Primers Pro (S4 and S5 Figs). When the concentration of YNFS Y Primers Pro was 1 μL, only 42.22% (19/45) of loci were successfully typed. Generally speaking, reasonable concentration variations of PCR components can not significantly affect the results of the kit.

### 3.2. Sensitivity studies

**Fig 3:**
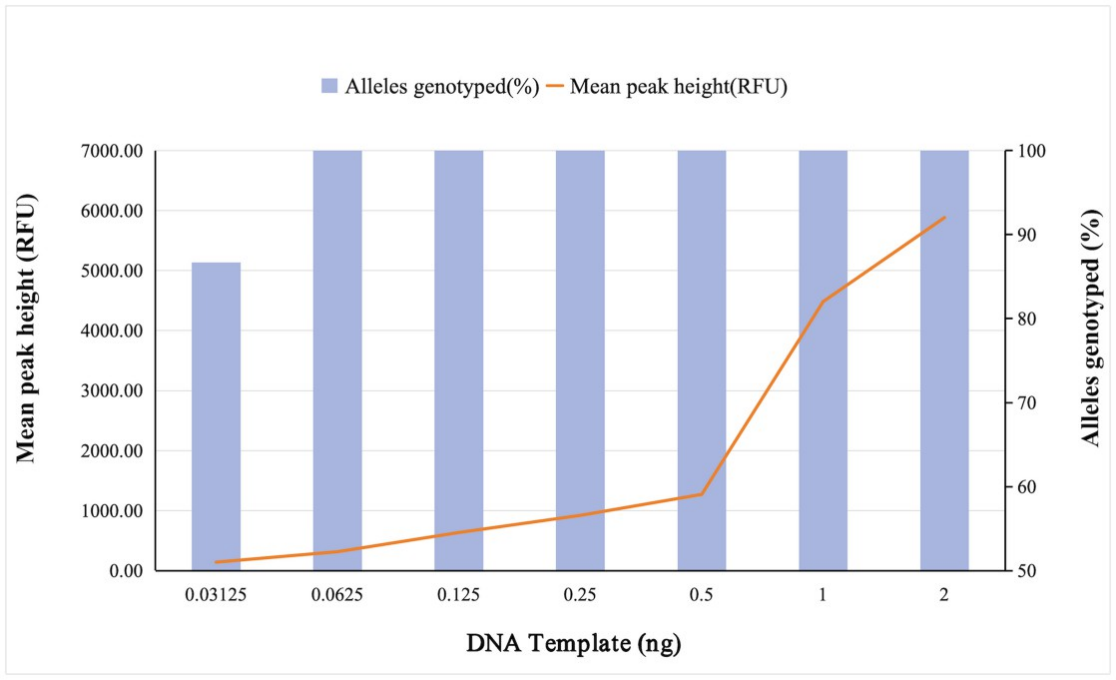
Mean peak heights and alleles genotyped of the sensitivity studies ranged from 0.03125 ng to 2 ng. The volume of PCR reaction was 25 μL.

There is a diversity of DNA samples in actual forensic cases, and trace DNA samples are common, often lower than the DNA concentration recommended by this system [22]. Therefore it is essential to explore the sensitivity of the kit to assess the range of applicable DNA quantities. A series of different control 9948 DNA inputs were detected in 25 μL reaction volume at an analytical threshold of 50 RFU (Fig 3, S6 Fig). The result showed that the lower the amount of DNA, the lower the peak heights. The mean peak heights ranged from 141.16 RFU to 5883.59 RFU, with a large increase at the DNA input of 1 ng. It could be observed that when the DNA input is as low as 0.0625 ng, the genotyping profile remains accurate and complete. Nonetheless, when the amount of DNA was 0.03125 ng, 86.67% of the loci could be detected with complete accuracy (Fig 3). Allelic loss was detected at the following five loci: DYS389I, DYS447, DYS389II, DYS19 and DYS627, and the allelic typing error has occurred at rs759551978. The study demonstrated that the optimal input of DNA in the AGCU YNFS Y Kit ranged from 0.0625 ng to 2 ng.

### 3.3. Species specificity studies and stability studies

On account of the complex composition of the samples obtained at the forensic scene and the possible presence of DNA from lots of other species, cross-reactivity testing was carried out using the DNA of nine animal species closely related to human activities (S7 Fig). No peaks above 300 RFU were detected in the ducks, rabbits, cattle, and cavies. A few significant peaks were detected in samples from dogs, cats, chickens, pigs, and goats, which could obtain allele genotyping. Among them, in the dog sample, the peak heights of alleles at DYS437, DYS481, and Y_GATA_H4 exceeded 300 RFU and up to 900 RFU. The fragments of amplification products detected were concentrated within 250 bp for the cat and an “OL” peak above 900 RFU was observed at DYS576 for the chicken sample. For pigs, at DYS635 and DYS443 we found alleles with peak heights greater than 500 RFU. Additionally, it’s worth noting that a bit more “OL” peaks and alleles were found in the sheep DNA sample and the peaks were more heterogeneous. These results were a reminder that special attention should be paid to samples with mixed DNA from more common poultry such as cats, chickens, pigs, and especially dogs and goats in practice.

In the practical application of forensic science, complex field environments, and diverse chemical elements are very common, which is likely to affect the analysis results of samples in the scene. Hematin reduces the activity of DNA polymerase, affecting PCR amplification efficiency through the function of fluorescence quenching [23, 24]. Humic acid is present in the soil and inhibits amplification by binding to DNA templates or directly affecting DNA polymerase activity. Tannic acid is also a relatively common inhibitor that affects PCR amplification efficiency. The results suggested that complete allele typing could be obtained at the hematin concentration up to 250 μM, humic acid concentration up to 250 ng/μL, or tannic acid concentration up to 50 ng/μL (S8 Fig). No alleles were obtained at hematin concentrations of 1000 μM or more, humic acid concentrations of 750 ng/μL or more, or tannic acid concentrations of 125 ng/μL or more. Only a few alleles were found at a hematin concentration of 500 μM, with allelic 19 at DYS622, allelic 16 at DYS557, allelic 21 and 22 at DYS527, allelic 14 at DYS443 and allelic 38 at DYS518. At a humic acid concentration of 500 ng/μL, allelic 16 at DYS557 and allelic 38 at DYS518 were observed. When tannic acid was 75 ng/μL, allelic 19 at DYS622, allelic 16 at DYS557, allelic 22 at DYS527, and allelic 38 at DYS518 were found; when tannic acid was 100 ng/μL, the last three loci mentioned above were still typed correctly. It was worth explaining that a high “OL” peak was usually seen at DYS391, probably generated by non-specific amplification of the primer itself. In review, the four loci, DYS622, DYS557, DYS527, and DYS518, were more likely to be properly typed. To summarize, the AGCU YNFS Y Kit showed some tolerance to different types and concentrations of inhibitors, and the effect of PCR inhibitors on amplification still required attention.

### 3.4. Mixture studies

**Fig 4:**
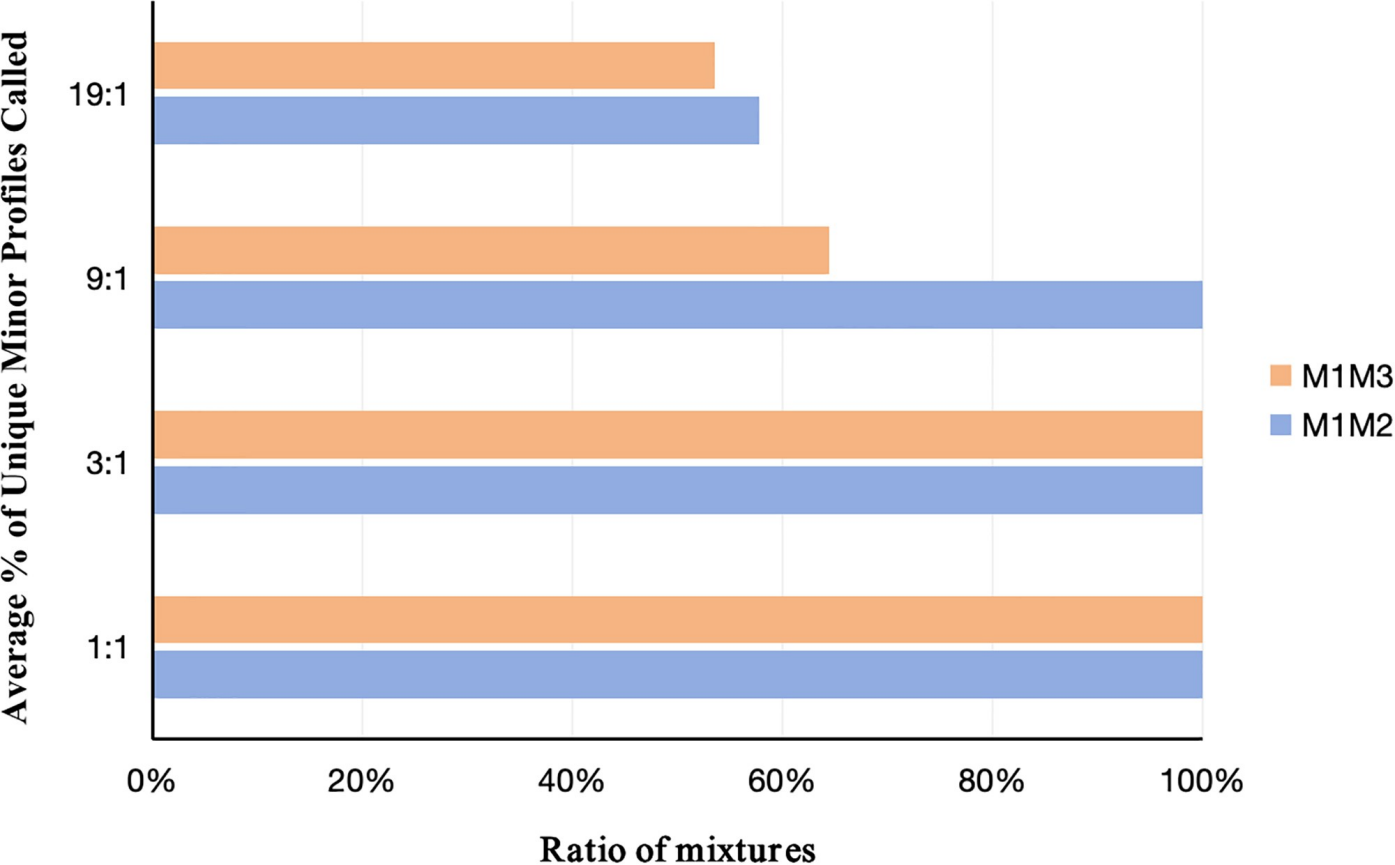
Amplification of two male/male/female mixtures. The total amount of DNA was 1ng for the male mixture samples and 400pg for the female. The mixing ratios were ranged from 1: 1 to 19: 1. M1M2 represented DNA 9948:M308 and M1M3 represented DNA 9948:007 in the figure.

It is more common for samples obtained in sexual assault cases to be either mixed male/female samples or multiple male mixed samples [25]. Based on this, the typing of different proportions of male mixtures in this kit was explored against a background of 400 pg of female DNA (S9 Fig). The main contribution was 9948 DNA, all of which could be detected. When the mixing ratio of 9948 DNA and M308 DNA was 19: 1, M308 DNA, which was a minor contributor, failed to obtain complete genotyping and the allele detection rate was 57.78% (Fig 4). The allele detection rates of 007 DNA were 53.55% and 64.44% respectively with the mixing ratios of 9948 DNA and 007 DNA being 19: 1 and 9:1. These outcomes indicated that this kit would give accurate and valid results when the mixtures were present in ratios greater than 9: 1.

### 3.5. Precision study and stutter calculation

**Fig 5:**
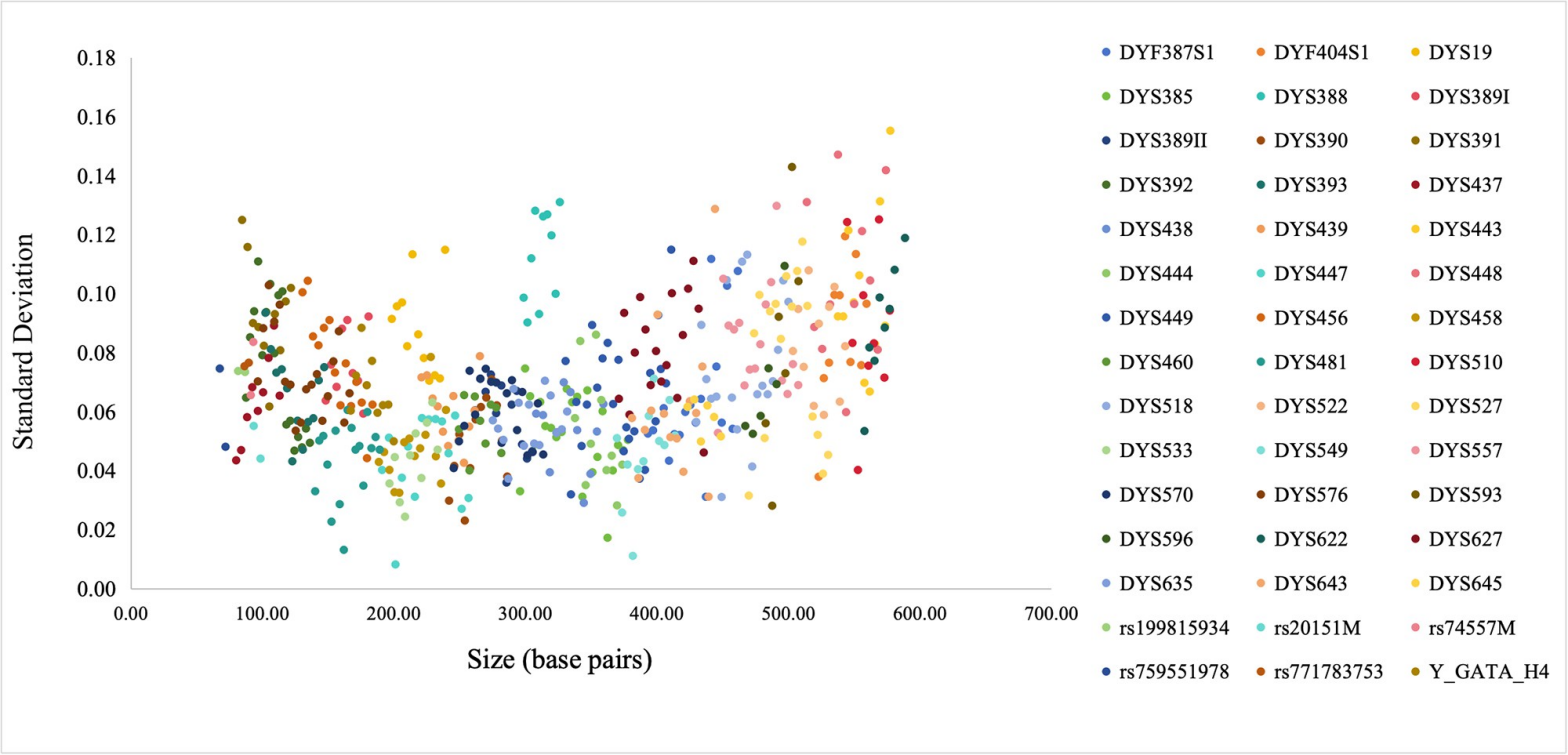
Precision across 24 injections of the AGCU YNFS Y Kit allelic ladder on an Applied Biosystems 3500 Genetic Analyzer.

Size precision is of paramount importance for reliable allele genotyping results. 45 allele ladders were detected on the 3500 Genetic Analyzer to calculate the average size and standard deviation. Scatter plots were generated by using average size as the horizontal coordinate and standard deviation as the vertical coordinate (Fig 5). The lowest standard deviation for DYS447 was 0.0083 and the highest standard deviation for DYS443 was 0.1552. It is demonstrated that the AGCU YNFS Y Kit was essentially free from the risk of genotyping errors due to insufficient size accuracy.

Stutter peaks are amplified fragments formed as a result of replication slippage during PCR amplification and tend to occur one repetition unit smaller before and after the target allele peak [26, 27]. A high proportion of stutter peaks can be easily confused with the typing of minor contributors, affecting the analysis of mixed samples [28]. In this study, a total of 23 loci had stutter peaks. The maximum stutter ratio, minimum stutter ratio, average stutter ratio, and standard deviation (SD) of all the loci with stutter peaks were counted (S1 Table). The lowest average stutter ratio was found at DYS622 (4.99%) and the highest at DYS570 (28.15%). The recommended stuttering filter threshold was set to the average stutter ratio plus three standard deviations [29], which was able to be applied in forensic practice.

### 3.6. Mutation analysis and population study

87 father-son pairs with confirmed parentage were used in the mutation analysis study. The electropherograms for one sample and the allelic ladder are shown in S10 and S11 Figs. Mutations occurred in 23 of the 87 father-son pairs, for a total of 26 mutation events. The number of father-son pairs in which 1, and 2 mutation events occurred was 20 and 3 pairs respectively. A total of 18 loci among the 45 loci were counted as mutated, with a range of mutation rates of 11.5×10^−3^–34.5×10^−3^, all larger than 1×10^−2^ (Table 1). Among the 18 loci that were mutated, five loci have been confirmed to be FM Y-STRs by previous studies [6, 7], including DYS627, DYF404S1, DYS576, DYS570, and DYF387S1. Intriguingly, in this study, the two loci with the highest mutation rates (34.5×10^−3^) were also DYS627 and DYF404S1, and their 95% CIs were both 7.2×10−3–97.5×10^−3^. This illustrated the strong potential of these two loci to differentiate between male relatives of the same paternity. The lowest mutation rate among the other loci was 11.5×10^−3^ with a 95% CI of 0.3×10−3–62.4×10^−3^. Among the mutation events, the numbers of extended and reduced mutations were twelve and fourteen, respectively, without significant bias, which was consistent with previous findings [30]. In addition, it was noteworthy that among these mutated loci, DYF404S1, DYS527, and DYF387S1 were all multicopy loci. Therefore, we hypothesized that the mutation rate of the loci tended to increase with increasing copy number.

**Table 1:** Number of mutations, number of mutation steps, number of gains and losses, mutation rates, and Binomial 95% CIs of 18 mutated loci.

Y-STR Loci	Number of mutations	Number of one-step mutations	Number of two-step mutations	Number of multi-step mutations	Number of gains	Number of losses	Mutation rates (×10^−3^)	Binomial 95% CIs (×10^−3^)
DYS627	3	3	0	0	2	1	34.5	7.2–97.5
DYF404S1	3	3	0	0	2	1	34.5	7.2–97.5
DYS447	2	2	0	0	2	0	23	2.8–80.6
DYS622	2	2	0	0	0	2	23	2.8–80.6
DYS527	2	2	0	0	1	1	23	2.8–80.6
DYS576	2	2	0	0	1	1	23	2.8–80.6
DYS389II	1	1	0	0	0	1	11.5	0.3–62.4
DYS549	1	1	0	0	1	0	11.5	0.3–62.4
DYS522	1	1	0	0	1	0	11.5	0.3–62.4
DYS460	1	1	0	0	1	0	11.5	0.3–62.4
DYS533	1	1	0	0	0	1	11.5	0.3–62.4
DYS510	1	1	0	0	0	1	11.5	0.3–62.4
DYS393	1	1	0	0	0	1	11.5	0.3–62.4
DYS439	1	1	0	0	0	1	11.5	0.3–62.4
rs74557M	1	1	0	0	0	1	11.5	0.3–62.4
DYS458	1	1	0	0	1	0	11.5	0.3–62.4
DYS570	1	1	0	0	0	1	11.5	0.3–62.4
DYF387S1	1	1	0	0	0	1	11.5	0.3–62.4

A total of 87 unrelated individuals were analyzed for genotyping results in the population study. 86 haplotypes were present in the 87 Han Chinese male samples, with FUH of 0.9884, HD of 0.9997, and DC of 0.9885. Specific genotyping and haplotype frequencies were shown in S2 Table. A total of 257 alleles were detected in the 45 loci of the kit, with allele frequencies ranging from 0.0057 to 0.9770 (S3 Table). These results indicated that the kit is a useful tool for analyzing forensic cases and database samples.

### 3.7. Case samples and degradation studies

Evaluating the genotyping efficiency of degraded samples helps assess the stability and efficiency of the kit [11]. We therefore tested different kinds of artificially degraded samples exposed to UV light to assess the performance of the AGCU YNFS Y Kit. The results showed that in both single male blood samples and mixed semen-vaginal secretion samples, all samples were fully typed even after exposure to UV-C light for up to 120 hours (S12 and S13 Figs). In other words, the duration of exposure to UV light did not affect the typing results of male blood samples and mixed semen-vaginal secretion samples. However, of the three single male saliva samples, complete genotyping was obtained only at 0 and 24 hours in one sample, and only at 0 hours in the other two samples, both with a typing detection rate of 95.56% at 24 hours. All three samples had a genotyping detection rate of less than 90% at 48 hours-120 hours (S14 Fig). In three samples of male saliva and female blood mixture, all three samples could be typed completely accurately at 0 and 24 hours, but after 48 hours of UV exposure, only one sample was typed completely, and the other two samples both had one locus dropped out. At 72 to 120 hours, only less than 85% of the loci were accurately detected in all three samples (S15 Fig). S16 Fig showed the genotyping detection rates for three sets of saliva samples, and three sets of male saliva and female blood mixtures.

These results show that the AGCU YNFS Y Kit is stable and performs well for degraded samples, especially for degraded samples of blood and semen, which are more stable compared to saliva. It is thought that saliva is less stable than blood and semen because collected saliva may contain fewer shed cells of the oral epithelium and the oral cavity has a richer microbial community, which may have an impact on the extraction of DNA [31]. Semen is more stable because the nuclear membrane proteins of spermatozoa are rich in disulfide cross-links, which are less likely to be damaged. In sexual assault cases, mixtures of male and female samples are usually collected, and these two mixtures were also intended to explore the forensic applications of the kit. In conclusion, we suggest that UV exposure has a greater effect on saliva and almost no effect on blood and semen samples. Accurate typing of semen and vaginal secretion mixtures also demonstrated the high accuracy of the kit in differentiating between male and female samples.

## 4. Conclusion

In general, the AGCU YNFS Y Kit is suitable not only for genealogical investigations of males from the same paternal line and paternity testing but also for investigations of male suspects in mixed samples of sexual assault and other criminal cases. Validation experiments were conducted to verify the performance of the kit from the following aspects: PCR amplification conditions, sensitivity, stability, species specificity, precision and accuracy, stutter calculation, mixture testing, mutation analysis, population study, and case samples and degradation studies. All the results of the study showed that the kit is a highly sensitive, accurate, stable, convenient, and informative kit. Therefore it can be applied in practice as a powerful complementary tool to the existing Y-STR panel and promote the construction of Y chromosome databases, which have high application value in forensic practice.

## Supporting information

S1 Table Stutter percentages were calculated for 23 loci. (XLSX)

S2 Table Haplotype information of the 87 samples with haplotype frequencies, FUH, HD, DC, GD. (XLSX)

S3 Table Allele frequency and gene diversity for 87 samples. (XLSX)

S1 Fig Genotyping profiles of control 9948 DNA amplified with different PCR reaction volumes (3.5 μL, 5 μL, 10 μL, 15 μL, 25 μL). (DOCX)

S2 Fig Genotyping profiles of control 9948 DNA amplified with different PCR cycles (22 cycles, 24 cycles, 26 cycles, 28 cycles, 30 cycles, 32 cycles, 34 cycles). (DOCX)

S3 Fig Genotyping profiles of control 9948 DNA amplified with different annealing temperatures (56 °C, 58 °C, 60 °C, 62 °C, 64 °C). (DOCX)

S4 Fig Genotyping profiles of control 9948 DNA amplified with different concentrations of YNFS Y Mix Pro (6 μL, 8 μL, 10 μL, 12 μL, 14 μL). (DOCX)

S5 Fig Genotyping profiles of control 9948 DNA amplified with different concentrations of YNFS Y Primers Pro (1 μL, 3 μL, 5 μL, 7 μL, 9 μL). (DOCX)

S6 Fig Genotyping profiles of control 9948 DNA amplified with different concentrations of template DNA (0.03125 ng, 0.0625 ng, 0.125 ng, 0.25 ng, 0.5 ng, 1 ng, 2 ng). (DOCX)

S7 Fig Genotyping profiles of DNA from common animals (duck, rabbit, cattle, cavy, dog, cat, chicken, pig, goat). (DOCX)

S8 Fig Genotyping profiles of control 9948 DNA amplified with different concentrations of inhibitors (humic acid, hematin, tannic acid). (DOCX)

S9 Fig Genotyping profiles of template DNA mixed with male DNA and male DNA (9948: M308, 9948: 007) under the background of 400pg female DNA (K562) at various ratios (1:1, 3:1, 9:1, 19:1). (DOCX)

S10 Fig Electropherogram of one sample under standard thermal cycling conditions. (DOCX)

S11 Fig Electropherogram of allelic ladder designed for the AGCU YNFS Y Kit. (DOCX)

S12 Fig Genotyping profiles of one degraded male blood sample under different UV-C light exposure times (0 hours, 24 hours, 48 hours, 72 hours, 96 hours, 120 hours). (DOCX)

S13 Fig Genotyping profiles of one degraded mixed semen-vaginal secretion sample under different UV-C light exposure times (0 hours, 24 hours, 48 hours, 72 hours, 96 hours, 120 hours). (DOCX)

S14 Fig Genotyping profiles of one degraded male saliva sample under different UV-C light exposure times (0 hours, 24 hours, 48 hours, 72 hours, 96 hours, 120 hours). (DOCX)

S15 Fig Genotyping profiles of one degraded male saliva and female blood mixture under different UV-C light exposure times (0 hours, 24 hours, 48 hours, 72 hours, 96 hours, 120 hours). (DOCX)

S16 Fig Alleles genotyped of three saliva samples, and three male saliva and female blood mixtures. (DOCX)
