# Supplementary figures and images for: Developmental validation of the AGCU YNFS Y Kit: A new 6-dye multiplex system with 44 Y-STRs and 5 Y-InDels for forensic application
Source: PLoS One. 2024 Aug 9;19(8):e0308535. doi: 10.1371/journal.pone.0308535 (PMC11315348; doi:10.1371/journal.pone.0308535)

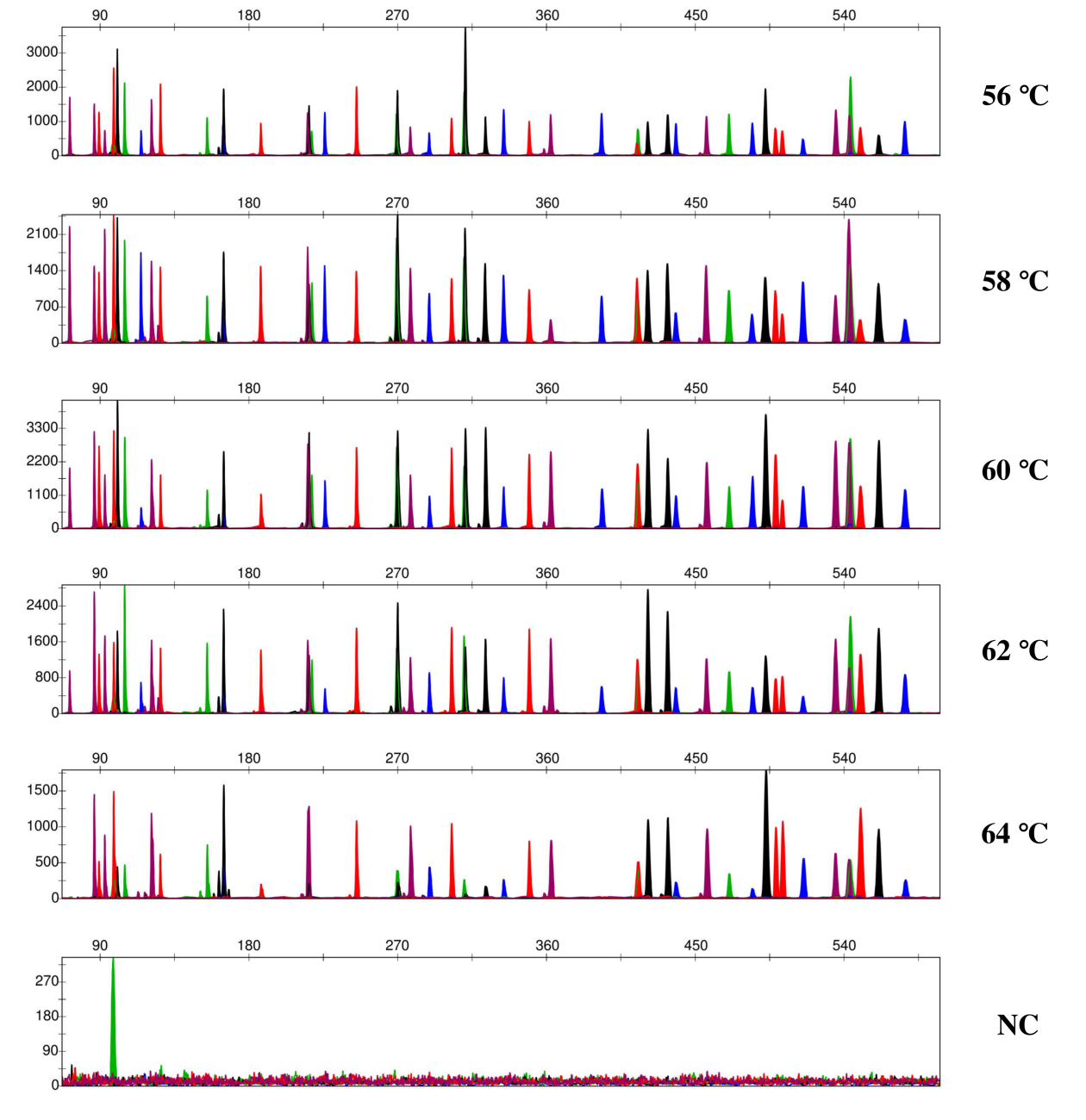


**Fig. S3** Genotyping profiles of control 9948 DNA amplified with different annealing temperatures (56 ℃, 58 ℃, 60 ℃, 62 ℃, 64 ℃)

Supplement: S3 Fig — (DOCX) [file pone.0308535.s006.docx]

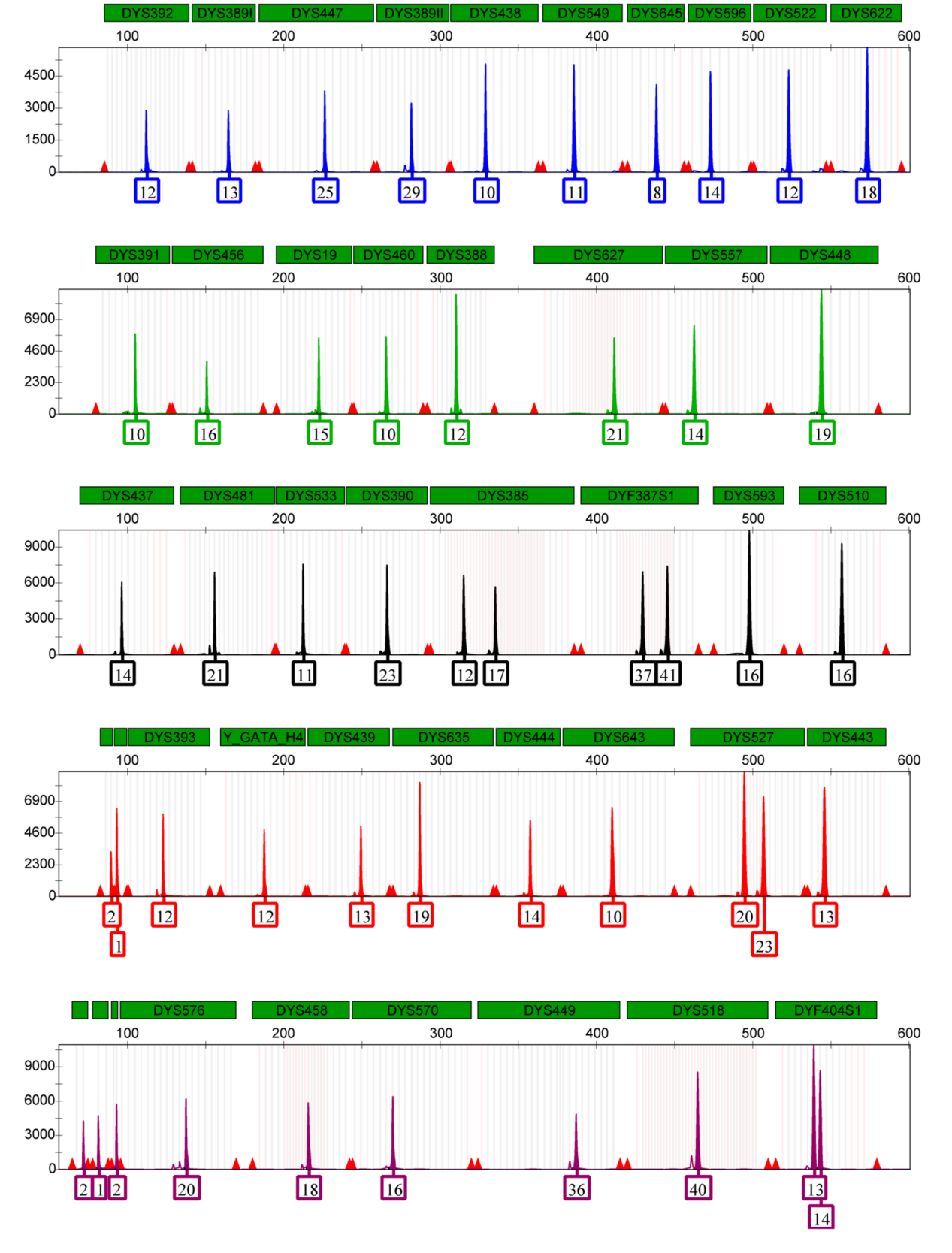


**Fig. S10** Electropherogram of one sample under standard thermal cycling conditions

Supplement: S10 Fig — (DOCX) [file pone.0308535.s013.docx]

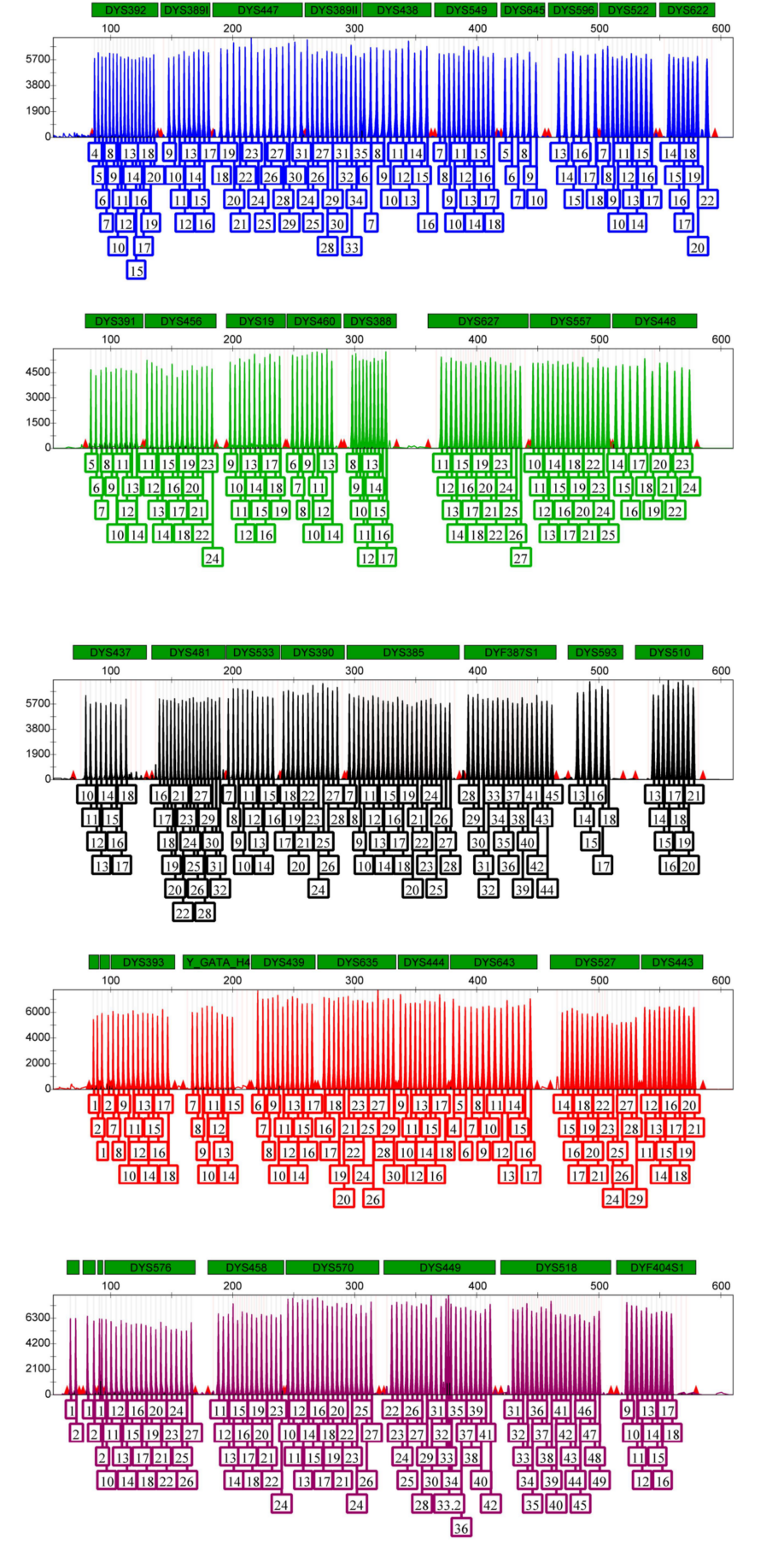


**Fig. S11** Electropherogram of allelic ladder designed for the AGCU YNFS Y Kit

Supplement: S11 Fig — (DOCX) [file pone.0308535.s014.docx]
