# Supplementary material for: Developmental validation of the AGCU YNFS Y Kit: A new 6-dye multiplex system with 44 Y-STRs and 5 Y-InDels for forensic application
Source: PLoS One. 2024 Aug 9;19(8):e0308535. doi: 10.1371/journal.pone.0308535 (PMC11315348; doi:10.1371/journal.pone.0308535)
Supplement: S5 Fig — (DOCX) [file pone.0308535.s008.docx]

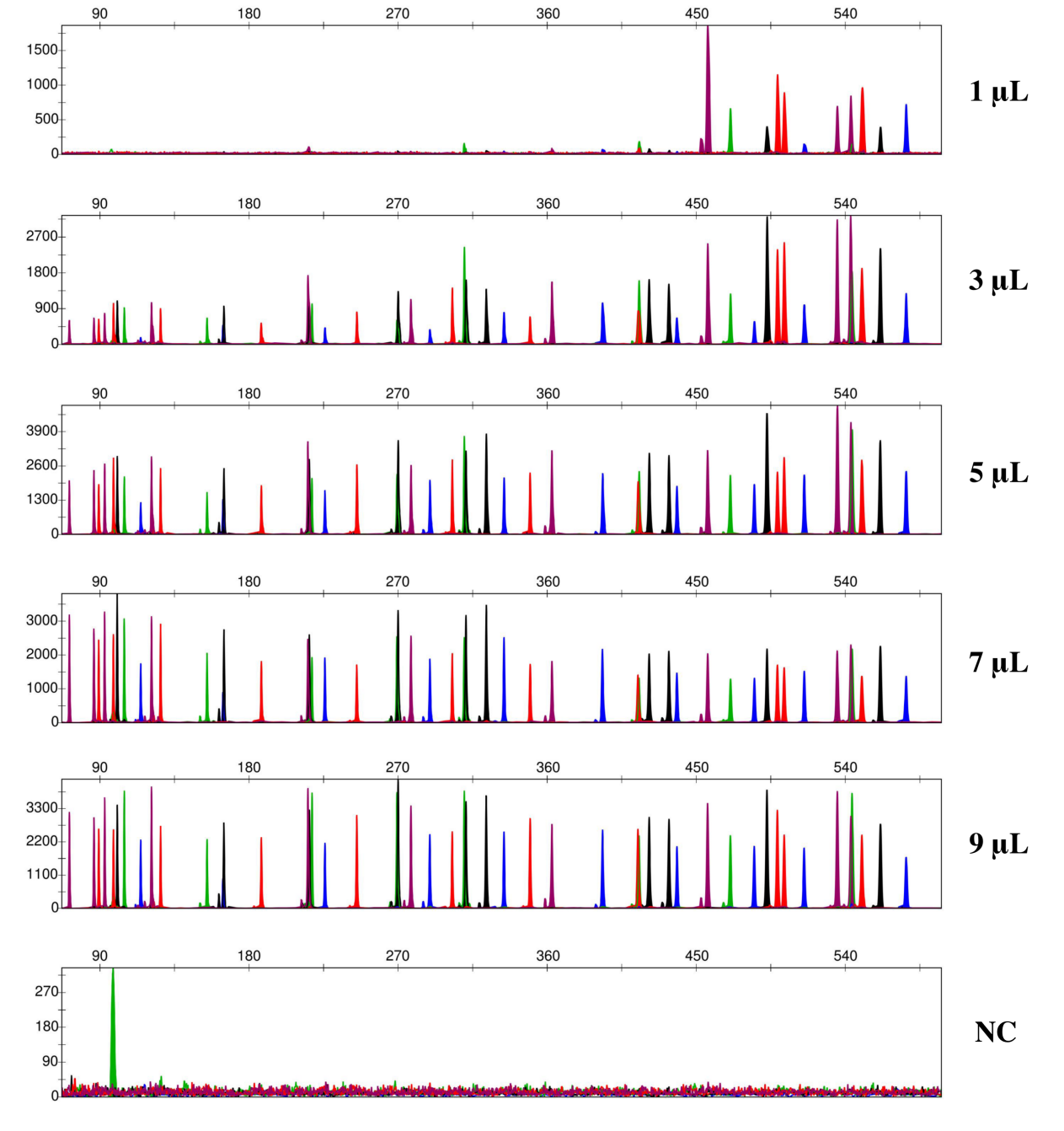


**Fig. S5** Genotyping profiles of control 9948 DNA amplified with different concentrations sof YNFS Y Primers Pro (1 µL, 3 µL, 5 µL, 7 µL, 9 µL)
