# Supplementary material for: Developmental validation of the AGCU YNFS Y Kit: A new 6-dye multiplex system with 44 Y-STRs and 5 Y-InDels for forensic application
Source: PLoS One. 2024 Aug 9;19(8):e0308535. doi: 10.1371/journal.pone.0308535 (PMC11315348; doi:10.1371/journal.pone.0308535)
Supplement: S6 Fig — (DOCX) [file pone.0308535.s009.docx]

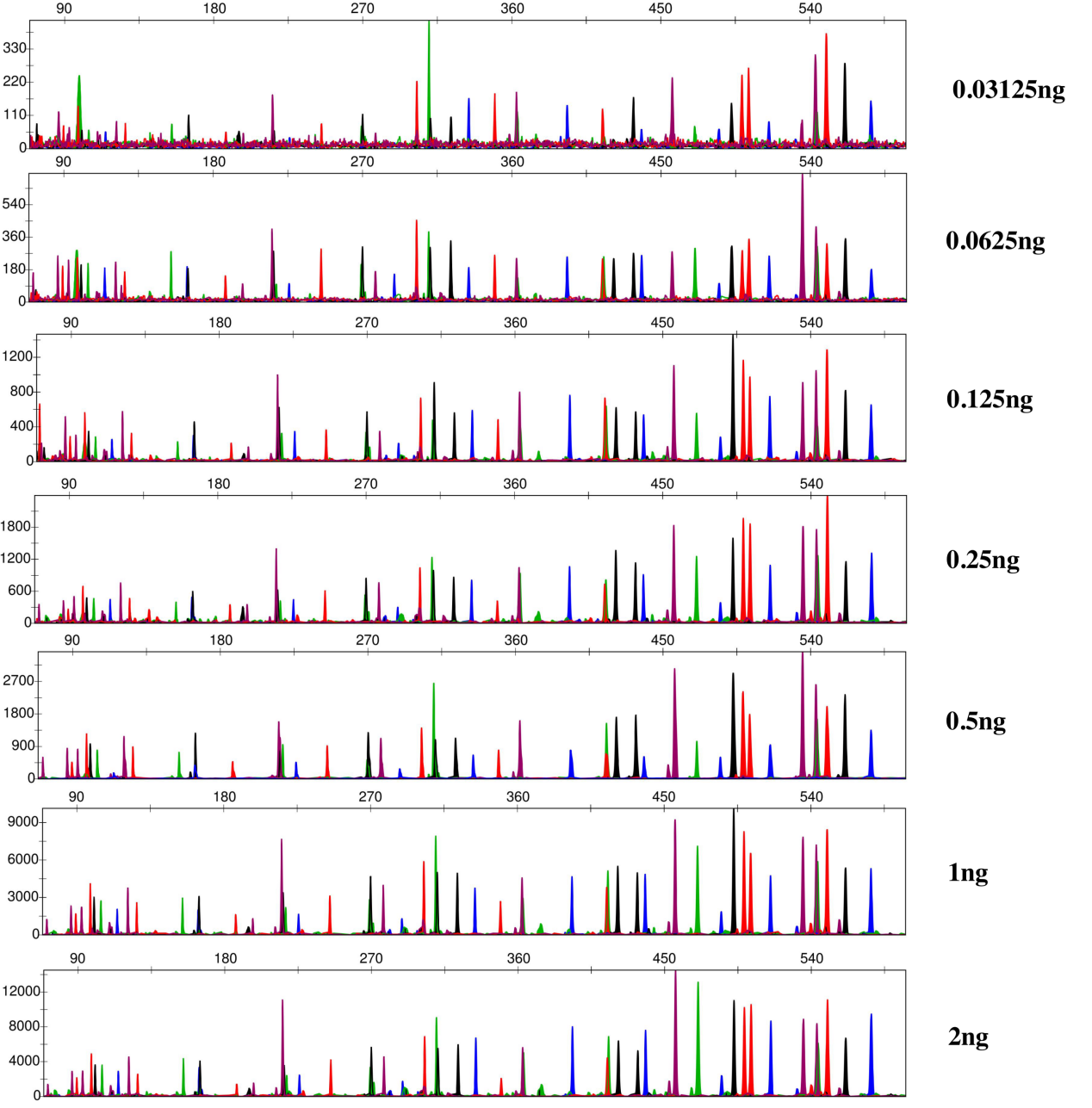


**Fig. S6** Genotyping profiles of control 9948 DNA amplified with different concentration of template DNA (0.03125 ng, 0.0625 ng, 0.125 ng, 0.25 ng, 0.5 ng, 1 ng, 2 ng)
