# Supplementary material for: Developmental validation of the AGCU YNFS Y Kit: A new 6-dye multiplex system with 44 Y-STRs and 5 Y-InDels for forensic application
Source: PLoS One. 2024 Aug 9;19(8):e0308535. doi: 10.1371/journal.pone.0308535 (PMC11315348; doi:10.1371/journal.pone.0308535)
Supplement: S9 Fig — (DOCX) [file pone.0308535.s012.docx]

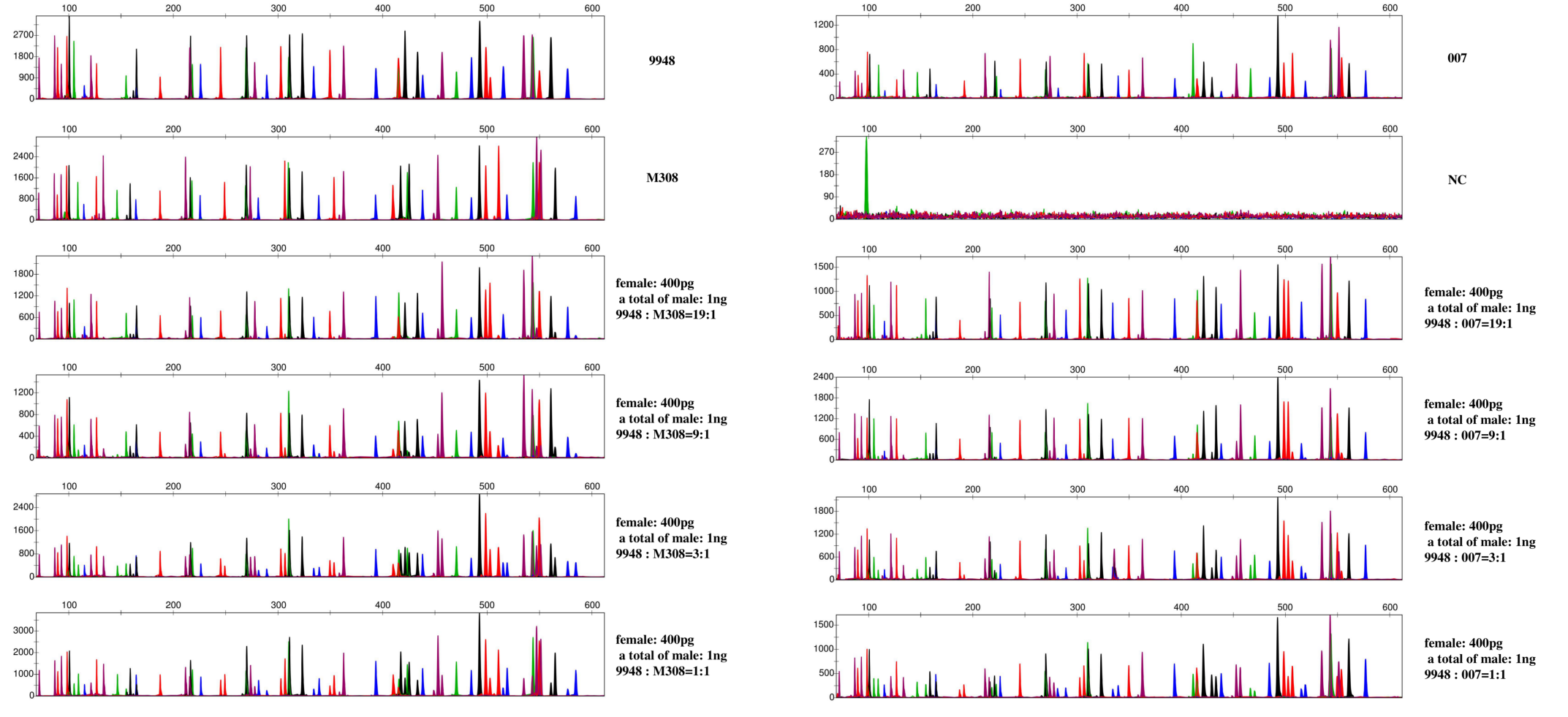


**Fig. S9** Genotyping profiles of template DNA mixed with male DNA and male DNA (9948 : M308, 9948 : 007) under the background of 400pg female DNA (K562) at various ratios (1:1, 3:1, 9:1, 19:1)
