# Supplementary material for: Developmental validation of the AGCU YNFS Y Kit: A new 6-dye multiplex system with 44 Y-STRs and 5 Y-InDels for forensic application
Source: PLoS One. 2024 Aug 9;19(8):e0308535. doi: 10.1371/journal.pone.0308535 (PMC11315348; doi:10.1371/journal.pone.0308535)
Supplement: S15 Fig — (DOCX) [file pone.0308535.s018.docx]

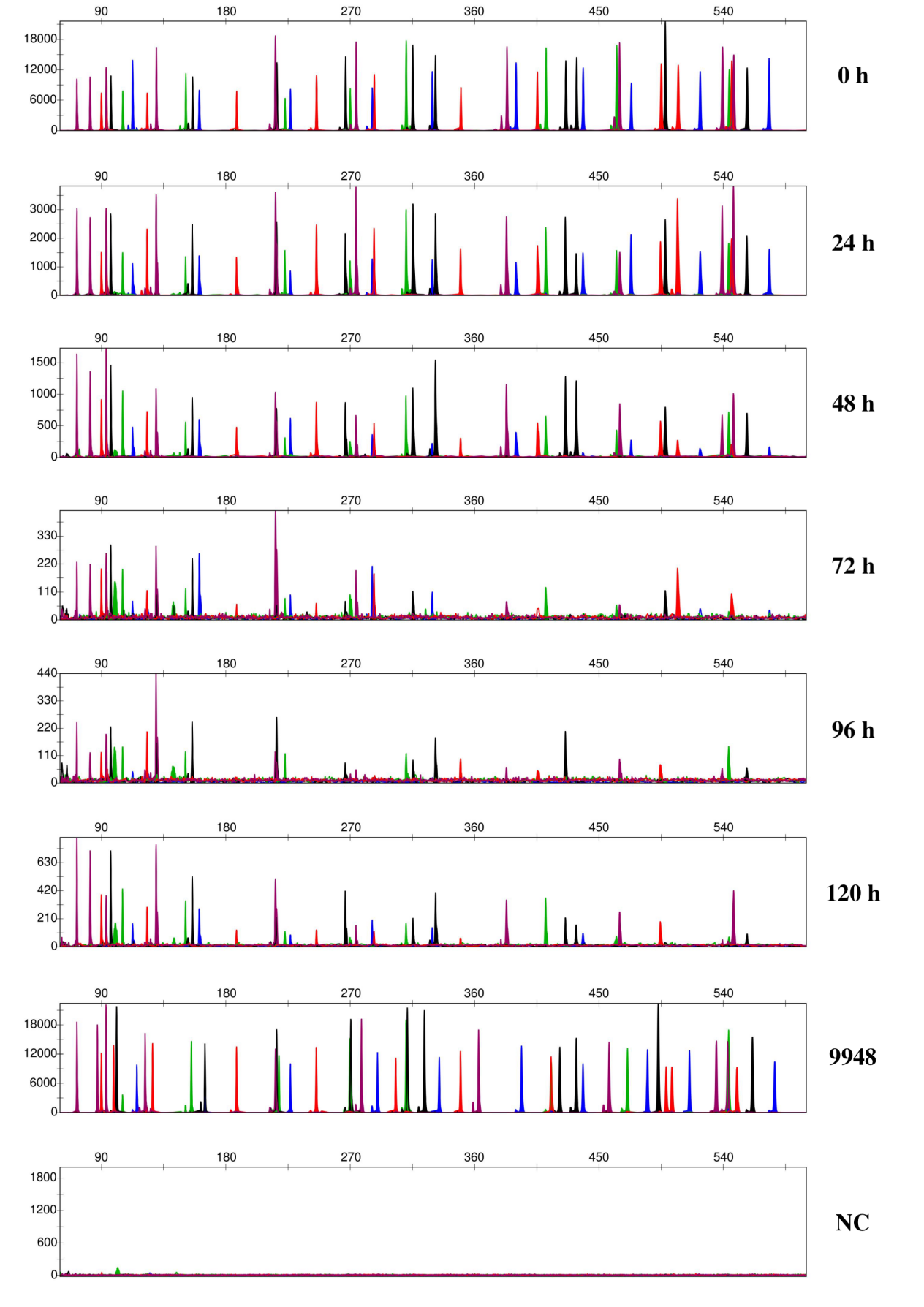


**Fig. S15** Genotyping profiles of one degraded male saliva and female blood mixture under different UV-C light exposure times (0 hours, 24 hours, 48 hours, 72 hours, 96 hours, 120 hours)
